# Novel Method for Sealing Tracheostomies Immediately after Decannulation—An Acute Clinical Feasibility Study

**DOI:** 10.3390/biomedicines12040852

**Published:** 2024-04-12

**Authors:** Rasmus Ellerup Kraghede, Karen Juelsgaard Christiansen, Alexander Emil Kaspersen, Michael Pedersen, Johanne Juel Petersen, John Michael Hasenkam, Louise Devantier

**Affiliations:** 1 Department of Anaesthesiology and Intensive Care, Aarhus University Hospital, 8200 Aarhus N, Denmark; 2 Department of Cardiothoracic and Vascular Surgery, Aarhus University Hospital, 8200 Aarhus N, Denmark; karen@clin.au.dk (K.J.C.); alemka@clin.au.dk (A.E.K.); hasenkam@clin.au.dk (J.M.H.); 3 Department of Clinical Medicine, Faculty of Health, Aarhus University, 8200 Aarhus N, Denmark; michael@clin.au.dk (M.P.); jojupe@rm.dk (J.J.P.); louisedevantier@clin.au.dk (L.D.); 4 Comparative Medicine Lab, Aarhus University, 8200 Aarhus N, Denmark; 5 Department of Otorhinolaryngology, Head and Neck Surgery, Aarhus University Hospital, 8200 Aarhus N, Denmark

**Keywords:** respiratory insufficiency, tracheostomy, clinical study, respiratory function tests, mechanical ventilators, rehabilitation, critical care

## Abstract

Tracheostomy decannulation leaves an iatrogenic passage in the upper airways. Inadequate sealing leads to pulmonary dysfunction and reduced voice quality. This study aimed to investigate the feasibility and impact of intratracheal tracheostomy sealing on laryngeal airflow and voice quality immediately after decannulation (ClinicalTrials.gov: NCT06138093). Fifteen adult, tracheostomized, intensive care unit patients were included from our hospital. A temporary, silicone-based sealing disc was inserted in the tracheostomy wound immediately after decannulation. Spirometry with measurement of forced vital capacity (FVC), forced expiratory volume in the first second (FEV_1_), and peak expiratory flow (PEF) were performed as measures of airway flow. Voice recordings were assessed using an equal appearing interval scale from 1 to 5. Median FVC, FEV_1_, PEF, and voice quality score with interquartile range (IQR) was 883 (510–1910) vs. 1260 (1005–1723) mL (*p* < 0.001), 790 (465–1255) vs. 870 (617–1297) mL (*p* < 0.001), 103 (55–211) vs. 107 (62–173) mL (*p* = 0.720), and 2 (1–2.5) vs. 4 (3–5) points (*p* < 0.001), respectively, with open tracheostomy vs. after sealing the tracheostomy with the intratracheal sealing disc. This feasibility study showed that tracheostomy sealing with the intratracheal disc was safe and led to immediate improvements in FVC, FEV_1_, and voice quality.

## 1. Introduction

Tracheostomy is the most common surgical procedure performed on critically ill patients in the intensive care unit (ICU) [1], and it is superior to oral intubation when prolonged mechanical ventilation exceeds 4–7 days [2,3]. Once weaning from mechanical ventilation is successful, continuous rehabilitation of pulmonary function is crucial [4,5]. However, the first 48 h following decannulation are particularly critical, and during this time interval, the risk of recannulation or reintubation is at its highest [6,7]. Decannulation failure is a well-documented complication, with failure rates reported as high as 25% [8,9,10].

Clinical indicators of effective decannulation include an undisturbed upper airway and the patient’s ability to remove pulmonary secretions [6]. Coughing, positive expiratory pressure (PEP) therapy, and application of intermittent continuous positive airway pressure (CPAP) are mechanisms that improve secretion clearance and aid pulmonary rehabilitation [11,12,13]. These mechanisms rely on an intact airway and act to pressurize the alveoli of the lungs to prevent the development of atelectasis and ensure sufficient gas exchange. However, loss of air through the tracheostomy wound may lead to insufficient restoration of pulmonary function caused by a cascade of diminished neuromuscular regeneration of the respiratory muscles, insufficient cough, accumulation of tracheobronchial secretion, atelectasis, and ultimately decannulation failure. Furthermore, the loss of air pressure hinders the patient’s ability to speak, having a significant impact on the overall wellbeing of patients following decannulation [14,15]. To counteract the loss of airway pressure through the tracheostomy wound, only simple, inefficient dressings or bandages are used in clinical practice [16]. These wound dressings are often loose or blow off during coughing or pulmonary exercises, and patients are instructed to apply pressure over the dressing manually [17], which is often a challenging task.

So far, primary focuses relied on the optimization of the pre-decannulation phase, such as weaning from ventilator therapy and monitoring dysphagia and cough strength prior to decannulation [18,19]. The post-decannulation phase is often neglected [20,21], and despite the fact that an effect of PEP therapy and CPAP on pulmonary rehabilitation has been demonstrated [22,23,24], no studies have investigated whether this effect is present in decannulated patients.

Recently, we presented a new concept that enables intratracheal sealing of the tracheostomy wound after decannulation by means of a sealing silicone disc placed on the luminal side of the trachea [25,26]. The hypothesis of our study is that sealing of the tracheostomy immediately after decannulation by insertion of the sealing disc re-establishes physiological airflow and improves the patient’s ability to speak compared with current standard care, leaving the tracheostomy wound open.

Accordingly, our aim was to investigate the feasibility of our novel concept for intratracheal sealing of the tracheostomy wound and to evaluate the immediate impact on physiological airway flow and voice quality.

## 2. Materials and Methods

### 2.1. Study Design, Setting, and Study Population

This feasibility study was performed in two steps. In sub-study one, a novel sealing disc prototype was produced and tested in two newly deceased human donors with no structural damage or previous major surgery of the upper airways. Sub-study two was a clinical, crossover feasibility study conducted on 15 subjects included from Department of Anaesthesiology and Intensive Care, Aarhus University Hospital, Aarhus, Denmark, from February 2020 to January 2022. Patients were eligible for inclusion when deemed ready for decannulation according to the following local clinical guidelines: (1) the patient must be awake, (2) the tracheostomy tube must have been capped for 24 h, (3) the need for suctioning airway secretion through the tracheostomy tube should not have exceeded two episodes within the past 24 h, (4) the patient must have sufficient cough ability, and (5) possible dysphagia should have been assessed. These guidelines are in line with decannulation practices worldwide [18]. Inclusion criteria included the following: age above 18 years, tracheostomy performed at least 7 days prior to decannulation, and the application of a tracheostomy tube with an inner diameter between 6–8 mm. Patients unable to cooperate or give informed consent were excluded.

### 2.2. Ethics

Sub-study one was acknowledged by Aarhus University, Aarhus, Denmark, as being in accordance with research allowed on deceased donors and required no additional ethical approval. Sub-study two was approved by the regional scientific ethics committee (Central Denmark Region, no. 1-10-72-112-19). Individual oral and written informed consent was collected from all subjects. The study is registered with ClinicalTrials.gov (NCT06138093).

### 2.3. The Tracheostomy Sealing Disc

**Figure 1:**
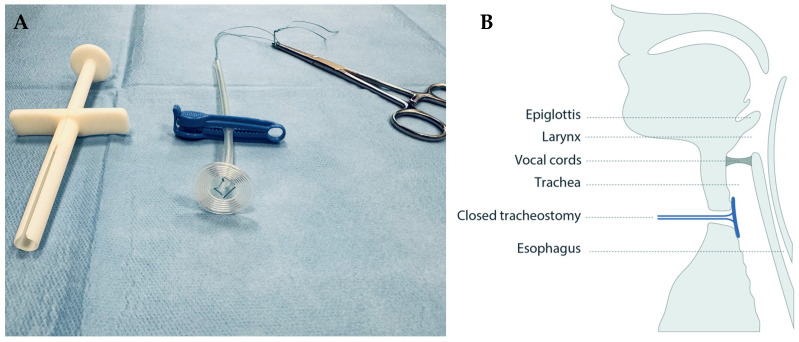
The tracheostomy sealing prototype (**A**) shown with a surgical suture imbedded in the clear silicone, a blue safety clamp locked around it, and a pean forceps placed in the suture after deployment, and (**B**) illustrated in situ in the tracheostomy, sealing it from the luminal side.

A circular sealing disc prototype made of medical grade silicone was produced in collaboration with engineers at Reenberg & Co (Graested, Denmark) and ProLink Sourcing and Trading (Macau, China). The prototype comprised three key components: a sealing disc, a rod acting as handle, and a braided polyester surgical suture imbedded in the silicone to which a clamp could be fastened for safety precautions (Figure 1A).

### 2.4. Procedure in Sub-Study One

A conventional surgical tracheostomy was performed on both cadavers. The sealing disc was inserted into the tracheal lumen through an insertion tube. In addition, oral intubation with an endotracheal tube of 7 mm allowed for the visualization of the silicone disc from the tracheal lumen using a regular aScope 4 Broncho bronchoscope (Ambu, Copenhagen, Denmark) with an outer diameter of 5 mm. The airways were pressurized via the endotracheal tube with a self-inflating Oval Silicone Resuscitator ventilation bag (Ambu, Copenhagen, Denmark) to simulate physiological airway pressure, while the sealed tracheostomy was monitored visually on the front of the neck for air leakage.

### 2.5. Procedure in Sub-Study Two

Each subject was seated in an upright position and decannulated. Local anesthesia (10% xylocaine spray) was sprayed into the tracheostomy wound. The sterilized sealing disc was sprayed with sterile saline water to lubricate and minimize friction and was inserted in the tracheostomy by means of a customized insertion tube (Figure 1B, Video S1). The rod and string of the sealing disc were anchored by two separate clamps outside the neck for safety precautions. A delicate pull on the sealing disc was maintained by hand for the full duration of the examination to keep the sealing disc in place. Finally, the sealing disc was removed by pulling the rod. Spirometry of exhaled air through the mouth and voice recordings were obtained when the tracheostomy was left open and after sealing by insertion of the sealing disc.

### 2.6. Data Collection

Spirometry data were collected using the Pneumotrac Spirometer with Spirotrac version 5 software (Vitalograph, Buckingham, UK), while voice recordings were performed using an iPhone 7 with Apple Voice Memos software (iOS version 14.1, Apple Inc., Cupertino, CA, USA). Spirometry was performed in accordance with guidelines on spirometry issued by the American Thoracic Society and European Respiratory Society [27]. For each subject, spirometry was performed with open tracheostomy and after insertion of the sealing disc. To ensure consistency in data and prevent tiredness or discomfort, each spirometry analysis was performed three times. Measures of forced vital capacity (FVC), forced expiratory volume in first second (FEV_1_), and peak expiratory flow (PEF) were obtained. According to logopedic consultation, the voice recordings were standardized by letting the subject count from one to ten and perform an “ah” sound for at least 3 s with the sound recorder placed approximately 50 cm from the face of the subject. Voice recordings were evaluated by two blinded evaluators using an equal appearance interval scale ranging between 1–5, where 5 represented normal voice quality and 1 represented a severely impaired voice [28,29]. Discrepancies between the two evaluations were solved by assessment from a third evaluator. Patient characteristics and measurement data were collected from patient files and managed using REDCap electronic data capture tools hosted at Aarhus University [30,31].

### 2.7. Statistical Analysis

Spirometry test data for each subject were pooled by group (open, sealed) by calculating the mean value of the three tests. Normality of continuous variables was assessed by inspection of quantile plots and tested with the Shapiro–Wilk test. For all spirometry parameters, logistic transformation improved normality while voice quality assessments were normally distributed. However, due to the limited sample size a conservative approach was chosen, and spirometry test data and voice quality assessments were presented as medians with interquartile range (IQR). Pairwise comparisons of all parameters between groups were done using the Wilcoxon signed rank test. All tests were 2-tailed and interpreted at a statistical significance level of 0.05. Statistical analyses were performed with support from the Biostatistical Advisory Service, Aarhus University, Aarhus, Denmark, using SAS Enterprise Guide software, version 7.1 (SAS Institute, Cary, NC, USA).

## 3. Results

In both human cadavers in sub-study one, the silicone disc could be introduced and deployed in an easy and simple maneuver. The endoscopic visualization showed smooth alignment of the disc to the inner surface of the trachea. After pressurizing the airways with a ventilation bag, only insignificant leakage was measured by external inspection of the tracheostomy.

All 15 subjects completed sub-study two. For practical logistic reasons, tests (spirometry and voice recordings for quality assessment) with the tracheostomy left open were made prior to temporary sealing of the tracheostomy with the sealing disc for six patients, while tests with the tracheostomy left open were performed after testing with the tracheostomy sealed with the disc in nine patients. This different succession was dictated by practical logistic handling of the patient. The deployment of the sealing disc was performed in less than a minute and was only associated with slight discomfort similar to suctioning through the tube, causing the subjects to cough for a few seconds. No subject experienced any complications with the sealing disc in place (severe discomfort, difficulty breathing, subcutaneous emphysema etc.).

### 3.1. Patient Characteristics

Patient characteristics are summarized in Table 1. Most subjects were admitted to the ICU due to respiratory failure. Several had comorbidities such as diabetes, chronic obstructive pulmonary disease, heart failure, and previous stroke. The subjects were hospitalized in the ICU for a mean of 10 days before being tracheostomized, most often using a percutaneous dilatational tracheostomy, and they were decannulated after a median of 15 days following tracheostomy. Median duration of stay at ICU (IQR) was 24 (19–30) days.

### 3.2. Spirometry

**Figure 2:**
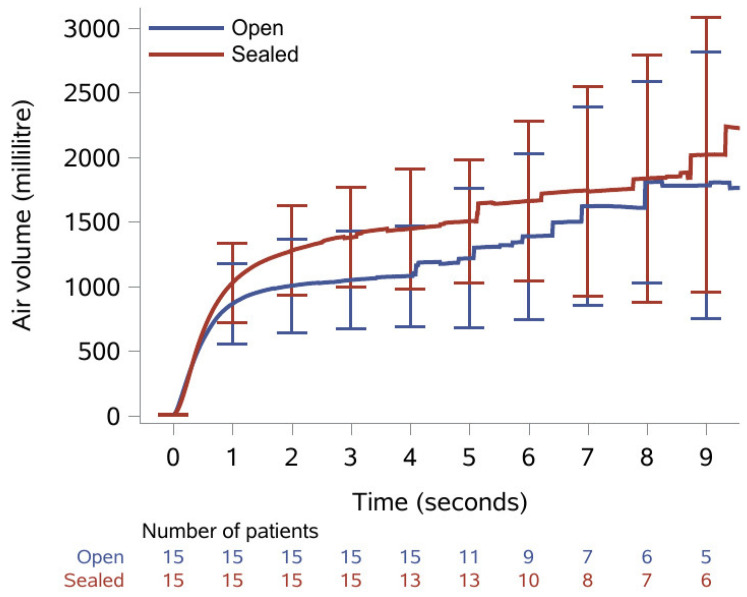
Mean exhaled volume during spirometry over nine seconds with 95% confidence intervals and number of patients remaining at each second with open tracheostomy (blue line) and when sealed with the sealing disc (red line).

The results of the spirometry recordings are presented as medians with IQR in Table 2. Median FVC was statistically significantly increased from 833 mL with open tracheostomy to 1260 mL after the tracheostomy was sealed with the sealing disc; a relative increase of 51%. Median FEV_1_ was 790 mL with open tracheostomy and statistically significantly increased to 870 mL when the tracheostomy was sealed; a relative increase of 10%. Median PEF was increased from 103 mL with open tracheostomy to 107 mL when sealed, yet this was not statistically significant. Additionally, Figure 2 shows the mean volume with confidence interval plotted as a function of time. All patients managed to exhale for ≥3 s, while only 10 patients (9 with open tracheostomy) managed to exhale for the 6 s recommended for a spirometry analysis.

### 3.3. Voice Quality Assessment

**Figure 3:**
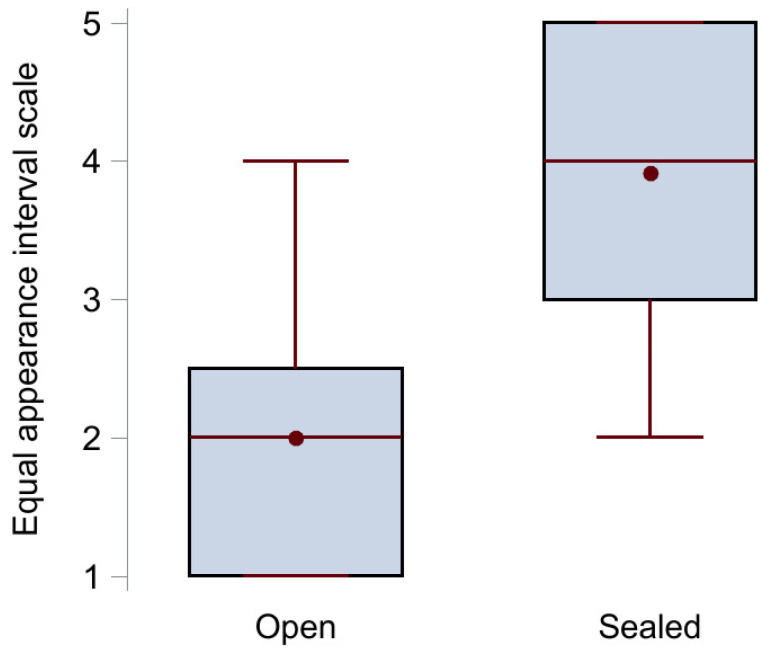
Box plot of the voice quality assessment results showing median (middle red line), mean (red circle), upper (75th percentile) and lower (25th percentile) quartile (black box), and maximum and minimum value (red whiskers) for the group with open tracheostomy (no sealing disc) and the group with sealed tracheostomy.

The results from the voice quality assessment using the equal appearance interval scale are illustrated in Figure 3. The median score (IQR) with open tracheostomy (n = 12) was 2 (1–2.5) points, while it statistically significantly increased to 4 (3–5) points when the tracheostomy was sealed (n = 11), *p* < 0.001. Examples of voice recordings with open (Audio S1A) and sealed (Audio S1B) tracheostomy are available as Supplementary Files.

## 4. Discussion

This study presents the first successful acute minimally invasive immediate sealing of tracheostomy after decannulation. The sealing disc was easily applied and removed first in two human cadavers and later in 15 patients after decannulation in the ICU. We observed no unintended adverse effects from the procedure. The positive impact in respiratory parameters and voice quality in sub-study two indicate that insertion of our sealing disc resulted in regained natural airflow through the larynx. The increase in measured FVC and FEV_1_ was to be expected when closing the tracheostomy. It does not necessarily reflect an increase in pulmonary capacity, since an evaluation of FVC and FEV_1_ as an indication of pulmonary function would require measurements of exhaled air through both the mouth and the tracheostomy. Yet, this study demonstrates a re-establishment of physiological airflow passing through the larynx. This enables the larynx to stop airflow and pressurize the thorax, which is necessary for sufficient coughing and pulmonary rehabilitation. In contrast to the application of a dressing over the tracheostomy (which may blow off during coughing) [17], the sealing disc successfully managed the stress of forced expiration without displacement or being blown out of the tracheostomy. This approach may provide a significant improvement in the post-cannulation treatment of tracheostomy wounds with respect to cognitive and physical performance for these critically ill patients.

When comparing our spirometry results with reference values, it is evident that our study population had very low values of FVC and FEV_1_ [32]. This finding highlights the importance of optimizing pulmonary physiology for this patient group, since previous studies on pulmonary function and mortality have shown that low FEV_1_ and FVC are associated with an increased mortality [33,34]. Surprisingly, PEF did not change significantly in this acute study, despite re-establishment of physiological airflow in the upper airways. Since poor coughing abilities and low PEF are correlated, we expected PEF to increase more.

From the present study, we have not proven whether tracheostomy sealing affects the impact of PEP therapy and CPAP post decannulation or whether air leakage through the tracheostomy is proportional to ineffective pulmonary rehabilitation. However, we believe that with a sealed tracheostomy, patients will be able to perform pulmonary exercises with CPAP and PEP therapy and gain similar effects as non-tracheostomized patients [12,24]. This can potentially improve the post-decannulation course for tracheostomized patients compared with current typical practices.

Our results also showed improved voice quality with tracheostomy sealing compared with open tracheostomy. It has been shown how the patient’s ability to speak affects patient satisfaction and patient care during hospitalization [14]. Sealing the tracheostomy wound is likely to enhance the patients’ ability to express needs and interact with family or hospital staff.

### 4.1. Limitations

Spirometry on airflow through the mouth was used as a surrogate marker for changes in physiological airflow. More advanced parameters and sophisticated assessment tools (measuring exhaled air through mouth and tracheostomy or intrathoracic pressure during coughing) might improve the quality of data related to air leakage through the tracheostomy and interpretations of the impact of sealing. Voice evaluation was focused on overall voice quality as a single parameter. More advanced phonetic tools could have been employed. For practical logistics, some patients performed measurements with open tracheostomy after the measurements with sealed tracheostomy were done. This could potentially have induced biases if the different number of patients in each group creates variability between groups. Lastly, the relatively small sample size reduces the generalizability and statistical power of the study. Performing a sample size calculation prior to inclusion was not possible due to the nature of the study. To our knowledge, no other published studies have evaluated similar interventions on an ICU population. The sample size was based on our expectations of the spirometry and voice quality results. However, in this stage of feasibility investigation, the sample size provides enough insight of the concept to verify that our intuitive expectations for the impact of the sealing were correct and that the approach was feasible. Investigating how this novel tracheostomy sealing impacts long-term pulmonary rehabilitation compared to conventional treatment will require a larger sample size.

### 4.2. Perspective

This study showed the feasibility of intratracheal tracheostomy sealing and the results supported its clinically relevance. We envision that the absence of air leakage through the tracheostomy wound will trigger a positive cascade of improved cough effectiveness, improved airway sanitation for removal of pulmonary secretions, increased neuromuscular regeneration of respiratory muscles, and improved pulmonary rehabilitation, concomitant with shortening the duration of stay in the ICU and decreasing the risk of decannulation failure. When ensuring airtight sealing of the tracheostomy to improve cough efficacy, this approach could become an integrated tool in the protocol of weaning from mechanical ventilation and could help reduce the weaning period. Additionally, we believe that reduced secretion and airflow through the tracheostomy wound will facilitate faster healing of the tracheostomy wound. Further research should investigate the benefits of this approach during the entire healing period of the tracheostomy. Extending the follow-up period allows for pulmonary rehabilitation to show in the spirometry results. Comparing the rate at which this rehabilitation occurs, between conventional and interventional treatment, would be intriguing. Adding advanced imaging techniques to examine the healing progress of the sealed tracheostomy and physiological monitoring of oxygen saturation, supplementary oxygen, respiratory frequency, etc., could further reveal the impact of the tracheostomy sealing. To conduct such a study, further development of the sealing disc is required. This development should include compatibility and safety testing of the prototype prior to evaluating it in a randomized controlled trial with several days of follow-up.

## 5. Conclusions

In this study, we demonstrated the feasibility and immediate advantages of intratracheal tracheostomy sealing. We have shown that the sealing disc is safe to apply. Sealing the tracheostomy resulted in an improved airflow through the upper airways by increasing FVC by 51% and FEV_1_ by 10% and improved voice quality by two score points. Thus, the intratracheal sealing disc has the potential to shorten and improve the post-decannulation phase for ICU patients.

## Figures and Tables

**Table 1:** Patient characteristics.

Characteristic	Number (%) or Mean ± SD ^a^ *n* = 15
**Demographics**	
Age (years)	60 ± 12
Sex (male)	10 (66.7)
BMI (kg/m^2^)	27.3 ± 5.1
Smoking	
Never	8 (53.3)
Previous	3 (20.0)
Current	4 (26.7)
Diabetes	12 (80.0)
COPD	13 (86.7)
Heart failure (EF < 45%)	13 (86.7)
Previous stroke	14 (93.3)
**Cause of admission**	
Respiratory failure	6 (40.0)
Cardiovascular disease	5 (33.3)
Sepsis	3 (20.0)
Trauma	1 (6.7)
**Time**	
LOS prior to ICU admission (days), median (IQR)	0 (0–4)
ICU admission until tracheostomy (days)	9.9 ± 4.0
Duration of mechanical ventilation prior to tracheostomy (days), median (IQR)	7 (6–11)
Duration of cannulation (days), median (IQR)	15 (9–20)
Duration of mechanical ventilation until decannulation (days), median (IQR)	22 (20–30)
**Tracheostomy characteristics**	
Type of tracheostomy	
PDT	12 (80.0)
CST	3 (20.0)
Cannula size (mm)	
6.0 mm	1 (6.7)
7.0 mm	2 (13.3)
8.0 mm	11 (73.3)
Missing	1 (6.7)

^a^ Data are presented as numbers with percentages or mean ± standard deviation if not otherwise specified. BMI: body mass index; COPD: chronic obstructive pulmonary disease; CST: conventional surgical tracheostomy; EF: ejection fraction; ICU: intensive care unit; IQR: interquartile range; PDT: percutaneous dilatational tracheostomy; SD: standard deviation.

**Table 2:** Spirometry results with open tracheostomy and after it was sealed by insertion of the sealing disc.

Parameters	Open	Sealed	*p*-Value
FVC (mL)	833 (510–1910)	1260 (1005–1723)	<0.001
FEV_1_ (mL)	790 (465–1255)	870 (617–1297)	<0.001
PEF (L/min)	103 (55–211)	107 (62–173)	0.720

Values are presented as median (interquartile range). FEV_1_: forced expiratory volume in the first second; FVC: forced vital capacity; PEF: peak expiratory flow.

## Data Availability

The datasets generated and analyzed during the study are available from the corresponding author upon reasonable request.

## Supplementary Materials

The following supporting information can be downloaded at: https://www.mdpi.com/article/10.3390/biomedicines12040852/s1, Video S1: Short video showing the loading of the silicone disc in the insertion tube and intratracheal insertion of the disc to seal the tracheostomy from the luminal side in a 3D printed phantom. Finally, the video shows an endoscopic view in a human cadaver of the deployment of the sealing disc in the trachea followed by removal. Audio S1: Voice recordings of a patient counting to ten in danish with (A) open tracheostomy and (B) sealed with the sealing disc.
